# MDR TB Transmission, Singapore

**DOI:** 10.3201/eid1907.120372

**Published:** 2013-07

**Authors:** Cynthia B.E. Chee, Li-Yang Hsu, Li-Hwei Sng, Yee-Sin Leo, Jeffery Cutter, Yee-Tang Wang

**Affiliations:** Tan Tock Seng Hospital, Singapore (C.B.E. Chee, Y.-S. Leo, Y.-T. Wang);; National University of Singapore, Singapore (L.-Y. Hsu);; Singapore General Hospital, Singapore (L.-H. Sng);; Ministry of Health, Singapore (J. Cutter)

**Keywords:** MDR TB, transmission, Singapore, foreign-born, institutional, HIV/AIDS, correctional, viruses, tuberculosis and other mycobacteria, TB

**To the Editor:** Over the past decade, the proportion of pulmonary multidrug-resistant tuberculosis (MDR TB) cases among Singapore-born patients remained low, whereas that among foreign-born patients was 10× higher (*1*,*2*). Since 2005, Singapore has experienced a sharp increase in the number of MDR TB cases from high-prevalence countries (*3*). We report local transmission of MDR TB in 2011, from a short-stay visitor to 2 Singapore-born persons in a correctional setting.

The index case-patient was a 34-year-old Burmese man (patient A) arrested 10 months after entering Singapore. A screening radiograph taken 2 days after arrest showed a right upper lobe cavitary lesion. The man was referred to the TB Control Unit. He had been coughing for 3 months but had no other concurrent conditions. When the abnormal radiograph results became known, the man was isolated within the prison. Sputum was collected, and first-line anti-TB drugs were administered pending sputum results. The sputum smear had 3+ acid-fast bacilli (AFB); mutations of the *rpoB* and *katG* genes were indicated by testing with GenoType MTDR*plus* (Hain Lifescience, Nehren, Germany). The patient’s treatment regimen was modified accordingly; appropriate second-line anti-TB treatment was started 14 days after he entered the institution. *Mycobacterium tuberculosis* complex (MTC) grew from sputum in 9 days; phenotypic drug-susceptibility testing (DST) demonstrated resistance to rifampin, isoniazid, streptomycin, and ethambutol and susceptibility to pyrazinamide, ethionamide, kanamycin, and ofloxacin.

One month after patient A was arrested, a Singapore-born man (patient B) in a public hospital received a diagnosis of HIV infection (67 CD4 cells/μL) and *Pneumocystis jirovecii* pneumonia. He was not an identified contact of patient A, although his job entailed accompanying prisoners from remand centers to justice courts. Antiretroviral treatment (ART) given 1 month after HIV diagnosis resulted in fever 7 days later. A repeat chest radiograph showed increased opacities in the left upper zone. Sputum smear was 4+ for AFB, and MTC with *rpoB* gene mutation was detected (Xpert MTB/RIF; Cepheid, Sunnyvale CA, USA). Second-line anti-TB drugs were administered. MTC was grown in sputum and blood in 14 and 32 days, respectively; phenotypic DST 8 weeks later showed a susceptibility profile that was identical (except for ethambutol susceptibility) to that of patient A.

Patient C was a 43-year-old Singapore-born man arrested 1 month after receiving an HIV diagnosis and beginning ART. He withheld his HIV status from prison authorities. He shared a cell with patient A for ≈48 hours at the remand center before the chest radiograph for patient A was taken. Patient C was released after 2 days and screened as a contact 2 months later; CD4 count was <20 cells/μL despite ART. Physical examination showed peripheral lymphadenopathy. T-SPOT.TB test (Oxford Immunotec Ltd., Abingdon, UK) result was negative, chest radiographs were unremarkable, and 2 sputum smears were negative for AFB (corresponding specimens for TB culture were negative). These findings were communicated to the patient’s primary physician. He was hospitalized 3 months later with fever and cough for 5 days but discharged himself, against medical advice, after 2 days. After 11 days, he was readmitted in septic shock to another hospital, at which time his sputum smear was positive for AFB and chest radiograph showed an increased right paratracheal stripe with right lower zone opacities. A bronchoesophageal fistula was also diagnosed, for which he declined intervention. Isoniazid, ethambutol, pyrazinamide, and rifabutin (his second-line ART regimen was incompatible with rifampin) were prescribed, and he discharged himself, against medical advice. After 5 days, he was readmitted with worsening cough; second-line anti-TB medications were instituted when his sputum specimen results were reported 8 weeks later as being phenotypically resistant to rifampin, isoniazid, and streptomycin. DST results for ethambutol were discrepant for isolates cultured from 2 sputum specimens and tested in 2 laboratories.

DNA genotyping by spoligotyping (Ocimum Biosolutions, Hyderabad, India) (*4*) showed that the isolates from all 3 patients belonged to type 467 H3, according to the SITVIT2 database (www.pasteur-guadeloupe.fr:8081/SITVITDemo/). Mycobacterial interspersed repetitive units–variable number tandem repeat genotyping with a 24-loci panel (Genoscreen, Lille, France) (*5*) similarly showed identical profiles (Figure). No other isolates in our database had matching profiles.

**Figure:**
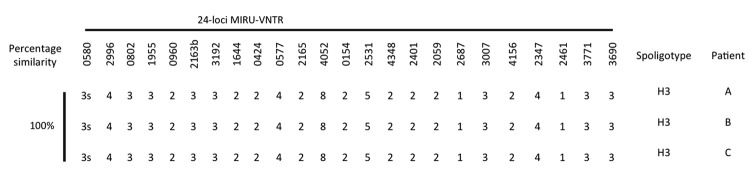
Spoligotype and 24-loci MIRU-VNTR typing results for *Mycobacterium tuberculosis* complex isolates recovered from 3 patients with multidrug-resistant tuberculosis (TB). Patient A (index case-patient), Burma-born man with TB, incarcerated in Singapore correctional facility; patient B, Singapore-born man with HIV infection and TB, who transported prisoners in Singapore; patient C, Singapore-born man with HIV infection and TB, who shared cell with patient A. MIRU-VNTR, mycobacterial interspersed repetitive units–variable number tandem repeat.

The cases reported here echo previous institutional outbreaks of MDR TB in industrialized countries (*6*–*8*). They are a reminder of the potentially devastating consequences when HIV and MDR TB intersect and the need for infection control measures where vulnerable and/or high-risk groups congregate. For patients A and B, rapid genotypic DST expedited the MDR TB diagnosis and institution of appropriate treatment and isolation measures and curtailed further spread. The unmasking immune reconstitution inflammatory syndrome that developed in patient B exemplifies the need for TB screening before starting ART in patients from countries with medium-to-high TB prevalence. For patient C, the several-week delay in instituting second-line TB medications could have been avoided had hospital medical teams been aware of his recent MDR TB exposure.

A recent update documented the highest rates of global MDR TB in 2009 and 2010 (*9*). Our experience reported here underscores the need to be constantly mindful of this infectious disease threat in our increasingly borderless world, even in countries where incidence of MDR TB is low.
